# Statin Use Is Associated with Decreased Risk of Invasive Mechanical Ventilation in COVID-19 Patients: A Preliminary Study

**DOI:** 10.3390/pathogens9090759

**Published:** 2020-09-17

**Authors:** Sophia L. Song, Sarah B. Hays, Constance E. Panton, Evangelia K. Mylona, Markos Kalligeros, Fadi Shehadeh, Eleftherios Mylonakis

**Affiliations:** Infectious Diseases Division, Warren Alpert Medical School of Brown University, Providence, RI 02903, USA; sophia_l_song@brown.edu (S.L.S.); sarah_hays@brown.edu (S.B.H.); constance_panton@brown.edu (C.E.P.); evangelia_mylona@brown.edu (E.K.M.); markos_kalligeros@brown.edu (M.K.); fadi_shehadeh@brown.edu (F.S.)

**Keywords:** COVID-19, SARS-CoV2, ICU, hospitalization, HMG-CoA reductase inhibitors, statins, invasive mechanical ventilation

## Abstract

COVID-19 disproportionately affects patients with medical comorbidities such as cardiovascular disease (CVD). Patients with CVD are widely prescribed 3-hydroxy-3-methyl-glutayl-CoA (HMG-CoA) reductase inhibitors (statins), a class of lipid-lowering medications known for their pleiotropic anti-inflammatory and immunomodulatory effects. However, the relationship between statin use and COVID-19 outcomes is not fully understood. In this preliminary study, we explored the association between statin use and severe COVID-19 outcomes in hospitalized patients, including intensive care unit (ICU) admission, the need for invasive mechanical ventilation (IMV), and in-hospital death. We performed a retrospective cohort study of 249 patients hospitalized with COVID-19 from 3 March 2020 to 10 April 2020 in Rhode Island, USA. Patient demographics, past medical history, current medications, and hospital course were recorded and analyzed. A multivariate logistic regression analysis was conducted to examine associations. After adjusting for age, sex, race, cardiovascular disease, chronic pulmonary disease, diabetes, and obesity, statin use was significantly associated with decreased risk for IMV (adjusted Odds Ratio (aOR) = 0.45, 95% Confidence Interval (CI): 0.20–0.99). Our results support the continued use of statins among COVID-19 patients and could have implications for future prospective studies on the management of COVID-19.

## 1. Introduction

Nearly five months after coronavirus disease 2019 (COVID-19), caused by severe acute respiratory syndrome coronavirus 2 (SARS-CoV2), was declared a pandemic by the World Health Organization, cases continue to rise across the world. The rapid spread of COVID-19 is especially alarming within the United States (US), which comprises 24.6% of the world’s cases as of 19 August 2020 [1].

Although COVID-19 manifests as a mild upper respiratory infection in most individuals, older adults with medical comorbidities are at the greatest risk of serious illness, leading to hospitalization and, eventually, death [2,3,4,5,6]. In particular, patients with underlying cardiovascular disease (CVD) may be disproportionately affected [7,8]. Moreover, CVD is the third most frequent comorbidity in COVID-19 patients, behind hypertension and diabetes [9,10]. These three comorbidities are closely related in terms of risk factors and underlying pathophysiology, including upregulation of the renin-angiotensin-aldosterone system, vascular injury, and inflammation [11].

3-hydroxy-3-methyl-glutayl-CoA (HMG-CoA) reductase inhibitors (statins) are a well-tolerated class of medications that are widely prescribed for the prevention of cardiovascular events, including stroke and myocardial infarction, in patients with atherosclerotic cardiovascular disease and hyperlipidemia [12,13,14]. While recent publications have investigated the potential for angiotensin-converting enzyme inhibitors (ACEis) and angiotensin II receptor blockers (ARBs) to exacerbate the course of COVID-19 infections [15,16,17,18,19,20], there is a paucity of literature regarding the influence of statins on COVID-19 infection outcomes.

Considering the relative safety of statins and high prevalence of CVD in COVID-19 patients, statins may be helpful in preventing subsequent cardiovascular complications associated with COVID-19, such as myocardial injury and heart failure [21]. Additionally, prior studies have demonstrated that statin usage is associated with improved outcomes in viral pneumonia and influenza [22,23,24,25]. Given that many COVID-19 patients already have primary indications for statins, further investigation into the relationship between statin use and COVID-19 outcomes may inform COVID-19 management.

In this study, we examined the relationship between commonly used chronic medications, including statins and ACEis/ARBs, and severe COVID-19 outcomes, including intensive care unit (ICU) admissions, invasive mechanical ventilation (IMV), and death, among patients hospitalized with COVID-19.

## 2. Results

We identified 249 adult patients who were admitted with COVID-19 to “Lifespan” healthcare system hospitals from 17 March 2020 to 10 April 2020. The baseline characteristics of these patients are described in Table 1. The median age of patients was 62 (IQR 51–75) years, 57% were men, 43.8% were non-Hispanic White, 36.5% were Hispanic, 14.9% were Black, and 0.4% were non-Hispanic Asian. The most common comorbidity was hypertension (49.0%), followed by obesity (40.2%), diabetes (33.3%), and heart disease (28.1%). The weighted Elixhauser score for this patient population was 4.12 (+/−7.78). For this patient population, 93 patients required ICU admission (37.3%), 45 patients required IMV (18.1%), and 42 patients died within the hospital (16.9%).

Statins were the most common medication class taken by patients on admission, with 49.4% of patients taking a statin. Other common medications included ACEis/ARBs (44.2%), beta blockers (30.1%), and oral antidiabetics (29.7%). The majority of statin users were White or Caucasian patients (52.0%) and greater than 65 years of age (64.2%). Statin users had a higher prevalence of hypertension (63.4%), diabetes (46.3%), and heart disease (37.4%) compared to non-statin users. The weighted Elixhauser score for statins users was higher at 5.18 (+/−8.60), compared to 3.09 (+/−6.75) for non-statin users. Statin users were also more likely to concomitantly take the following classes of medications: ACEis, ARBs, beta blockers, calcium channel blockers, diuretics, insulin, and oral antidiabetics (Table 1).

In the age-adjusted, age-, sex-, and race-adjusted and “fully adjusted” (adjusted for age, sex, race, cardiovascular disease, chronic pulmonary disease, diabetes, obesity) models, there were no significant associations between statin use and in hospital death or ICU admission (Table 2). In the age-adjusted and age-, sex-, and race-adjusted models, there were no significant associations between statin use and risk for IMV (aOR = 0.61, 95% CI: 0.29–1.24 and aOR = 0.57, 95% CI: 0.27–1.19, respectively). However, in the fully adjusted model, there was a statistically significant association between statin use and decreased risk for IMV (aOR = 0.45, 95% CI: 0.20–0.99, *p* = 0.048). A Mann–Whitney–Wilcoxon test showed no statistically significant associations between statin use and length of ICU stay or days on IMV (Supplementary Table S1). Additionally, a multivariable logistic regression analysis showed that ACEi or ARB use was not significantly associated with in-hospital death, ICU admission, or IMV in the age-adjusted, age-, sex-, and race-adjusted, or fully adjusted models (Table 3). A Mann–Whitney–Wilcoxon test showed no statistically significant associations between ACEi/ARB use and length of ICU stay or days on IMV (Supplementary Table S2).

## 3. Discussion

We report the results of a preliminary retrospective cohort study investigating associations between commonly used chronic medications, including statins and ACEis/ARBs, and severe COVID-19 outcomes among patients hospitalized with COVID-19, including ICU admission, invasive mechanical ventilation, and in-hospital mortality. We found that, in our fully adjusted model, statin use was significantly associated with decreased risk for IMV among patients hospitalized with COVID-19.

In the US, 12.1% of adults have a diagnosis of cardiovascular disease [26] and more than 26% of adults over age 40 take a statin [27]. Given the rapidly increasing rates of COVID-19 infections in the US, in the setting of a high national prevalence of CVD, our results have important implications for the management of COVID-19 in the US.

The prior literature discussing the effect of statins on respiratory illness has been inconclusive [22,23,24,25,28,29,30,31,32,33,34,35,36]. Statin use has been shown to protect against hospitalization for pneumonia and all-cause mortality in patients with seasonal influenza [22,23,24]. Furthermore, statin use may reduce the risk of complications of sepsis, acute lung injury, and acute respiratory distress syndrome (ARDS) in seasonal influenza [25,32]. Outpatient statin use has been associated with reductions in lung injury scores for patients infected with H1N1 influenza A virus [28,34]. The role of inpatient use of statins for pneumonia treatment is controversial, with several large-scale studies finding no effect of statins on mortality [33,36], while other studies claim lower risk of ICU admission, mechanical ventilation, and mortality for patients who continue statin use throughout hospitalization [30,31]. The potential benefit of statin use in patients with other forms of respiratory illness supports a possible role for statins in the treatment of COVID-19.

Several recent articles have proposed mechanisms for the role of statins in the treatment of COVID-19 and subsequent health outcomes in COVID-19 patients [35,37,38,39,40,41]. Statins have been shown to have anti-inflammatory effects through a reduction in C-reactive protein [42], tumor necrosis factor-alpha (TNF-alpha), and interferon-gamma [43,44] levels. However, other studies have suggested statins may block toll-like receptor and NF-κB signaling, thus increasing compensatory immune response and leading to adverse host outcomes [38]. Additionally, statins may upregulate the inflammatory cytokine IL-18, leading to tissue destruction through cytokine storm [35,40].

On the other hand, some investigators have advocated for the use of statins as a treatment for COVID-19, citing their cardio-protective effects, potential to improve endothelial function [45,46], and upregulation of the ACE2 receptor to reduce lung injury from dysregulated ACE2 levels [37,39,47]. Indeed, it has been demonstrated that higher levels of ACE2 lower severity of ARDS by countering endothelial dysfunction [48,49]. Furthermore, an in silico study found that statins bind to and inhibit the SARS-Cov2 main protease, disrupting viral maturation [41].

Our finding that statin use is associated with lower risk of IMV in COVID-19 patients is consistent with recent studies suggesting that statin use may improve COVID-19 outcomes. A retrospective study of patients across 21 hospitals in Hubei Province, China found that statin use was associated with decreased 28-day all-cause mortality [50]. Another retrospective study found that inpatient atorvastatin treatment was associated with a decreased risk of ICU death [51]. Moreover, statin use is associated with an absence of COVID-19 symptoms and trends toward improved clinical outcomes in nursing home residents [52].

There is great need for prospective studies investigating the effect of statins on COVID-19 outcomes. More data are needed to assess whether statin use is associated with improved outcomes other than IMV, as well as among outpatient COVID-19 patients or those without other indications for statin use. Several randomized control studies investigating these questions have been registered and are currently in progress. These include a phase 2 clinical trial exploring atorvastatin as an adjunctive treatment for COVID-19 [53], a phase 3 clinical trial investigating the effects of simvastatin, aspirin, and losartan combinations on COVID-19 mortality [54], and a phase 3 clinical trial examining the effects of atorvastatin treatment on venous thromboembolism (VTE), the need for extracorporeal membrane oxygenation (ECMO), and all-cause mortality in COVID-19 patients [55].

A strength of this study is that our fully adjusted model adjusted for multiple factors frequently associated with the severity of COVID-19 infections, including age, sex, race, and the common medical comorbidities of diabetes, cardiovascular disease, chronic pulmonary disease, and obesity. These have all been reported among the top five comorbidities most commonly associated with COVID-19 [2,3,4,5,6,56,57]. Another strength is that we reviewed consecutive patients admitted to our hospitals rather than selecting patients for inclusion, providing a realistic demographic and clinical picture of patient history and outcome. Although our retrospective study design is limited to estimating associations, our study does indicate certainty regarding the temporal sequence of exposure and outcomes.

Our study has several limitations that must be considered. First, the retrospective cohort design means that other potential sources of confounding cannot be excluded, and we cannot infer causality between statin use and decreased rates of IMV. Furthermore, we analyzed statin usage as a single medication class, leaving the possibility of different therapeutic effects based on statin type, strength, and dosage. Additionally, patient medication data were drawn from the electronic health record; as such, patients’ medical history may be incomplete, and researchers were unable to confirm that admitting providers fully reviewed admission medications with patients and patient pharmacies due to the high volume of patients seen daily. Finally, our cohort of patients with COVID-19 from a single state in the US may not reflect the health outcomes of COVID-19 patients nationwide.

In summary, our data support the continued use of statins among patients hospitalized with COVID-19, as continued statin usage is associated with decreased risk for IMV. Further studies are necessary to investigate whether de novo prescriptions of statins affects COVID-19 outcomes, regardless of other indications patients may have for statin administration. Given the wide availability and low cost of statins, there will be vast public health implications for COVID-19 management if further studies demonstrate similar evidence of improved outcomes among statin users.

## 4. Materials and Methods

### 4.1. Study Design and Patient Selection

Our study population included consecutive adult (≥18 years old) patients who were admitted with laboratory confirmed SARS-CoV-2 infection (using a reverse transcriptase polymerase chain reaction assay) to the “Lifespan” healthcare system (i.e., Rhode Island Hospital, The Miriam Hospital, and Newport Hospital, all in located Rhode Island, USA) from 17 March 2020 to 10 April 2020. This study was a retrospective electronic chart review, approved by the Institutional Review Board of the Rhode Island Hospital.

### 4.2. Data Collection

Six researchers (MK, FS, EKM, CEP, SBH, SLS) independently extracted demographic, epidemiological, clinical, and laboratory data of interest via manual review of patient electronic health records. Specifically, the following variables were extracted: age, race, sex, body mass index (BMI), past medical history, patient comorbidity (using van Walraven’s modification of the Elixhauser comorbidity index [58]), hospital course, and chronic medications (statins, angiotensin converting enzyme inhibitors, angiotensin II receptor blockers, beta blockers, calcium channel blockers, diuretics, insulin, and oral antidiabetics). Data were entered into the database organization software REDCap (Vanderbilt University, Nashville, TN, USA) and de-identified by assigning an ID number to each patient.

### 4.3. Study Outcomes

The outcome measures of our study were ICU admission, the need for IMV, and in-hospital death among patients hospitalized with COVID-19.

### 4.4. Statistical Analysis

Continuous and categorical variables were represented as medians (IQRs) and frequencies (%) and compared using the Mann–Whitney–Wilcoxon test and the Pearson’s chi-squared test, respectively. We conducted multivariate logistic regression analyses to examine associations between statin use and the outcomes of ICU admission, the need for IMV, and in-hospital death. Our analyses included adjustments for (1) age alone, (2) age, sex, and race, and (3) age, sex, race, and four common comorbidities (“fully adjusted model). The four comorbidities included cardiovascular disease (valvular disease, cardiac arrythmias, congestive heart failure, peripheral vascular disorders, hypertension with or without complications, pulmonary circulation disorders), chronic pulmonary disease, diabetes, and obesity. We adjusted for these comorbidities because hypertension, obesity, diabetes, and cardiovascular disease were the most common medical comorbidities within our patient population. Additionally, these comorbidities have been reported as among the most common comorbidities associated with COVID-19 [2,6,56,57]. For our analyses, 95% confidence intervals (CI), the standard deviation (SD), and p-values are shown. The statistical significance threshold was set at 0.05. All analyses were performed using Stata v15.1 (Stata Corporation, College Station, TX, USA).

## Figures and Tables

**Table 1:** Patient characteristics by statin use. *

	Total	No Statin Use	Statin Use	*p*-Value
	*n* = 249	*n* = 126	*n* = 123	
**Age ^†^**	62.0 (51.0–75.0)	54.5 (42.0–67.0)	71.0 (60.0–79.0)	<0.001
**Age group**				<0.001
**18–49 (*n* = 58)**	40.50 (31.00–44.00)	49 (38.9%)	9 (7.3%)	
**50–64 (*n* = 77)**	58.00 (54.00–61.00)	42 (33.3%)	35 (28.5%)	
**≥65 (*n* = 114)**	77.00 (71.00–83.00)	35 (27.8%)	79 (64.2%)	
**Race**				0.10
**Asian**	1 (0.4%)	1 (0.8%)	0 (0.0%)	
**Black or African American**	37 (14.9%)	22 (17.5%)	15 (12.2%)	
**Hispanic or Latino**	91 (36.5%)	51 (40.5%)	40 (32.5%)	
**Other/Unknown**	11 (4.4%)	7 (5.6%)	4 (3.3%)	
**White or Caucasian**	109 (43.8%)	45 (35.7%)	64 (52.0%)	
**Hypertension**	122 (49.0%)	44 (34.9%)	78 (63.4%)	<0.001
**Cardiovascular disease ^‡^**	70 (28.1%)	24 (19.0%)	46 (37.4%)	0.001
**Chronic pulmonary disease**	40 (16.1%)	14 (11.1%)	26 (21.1%)	0.031
**Diabetes**	83 (33.3%)	26 (20.6%)	57 (46.3%)	<0.001
**Renal failure**	25 (10.0%)	8 (6.3%)	17 (13.8%)	0.050
**Liver disease**	12 (4.8%)	6 (4.8%)	6 (4.9%)	0.97
**Obesity**	100 (40.2%)	48 (38.1%)	52 (42.3%)	0.50
**Weighted Elixhauser score (van Walraven)**	4.12 (7.78)	3.09 (6.75)	5.18 (8.60)	0.034
**Chronic Medications**				
**ACEis**	69 (27.7%)	26 (20.6%)	43 (35.0%)	0.012
**ARBs**	41 (16.5%)	12 (9.5%)	29 (23.6%)	0.003
**Beta Blockers**	75 (30.1%)	20 (15.9%)	55 (44.7%)	<0.001
**Calcium Channel Blockers**	63 (25.3%)	19 (15.1%)	44 (35.8%)	<0.001
**Diuretics**	55 (22.1%)	17 (13.5%)	38 (30.9%)	<0.001
**Insulin**	41 (16.5%)	12 (9.5%)	29 (23.6%)	0.003
**Oral antidiabetics**	74 (29.7%)	25 (19.8%)	49 (39.8%)	<0.001

* ACEis refers to angiotensin converting enzyme inhibitors. ARBs refers to angiotensin II receptor blockers; ^†^ reported as median (IQR); ^‡^ cardiovascular disease: valvular disease, cardiac arrythmias, congestive heart failure, peripheral vascular disorders, hypertension with or without complications, pulmonary circulation disorders.

**Table 2:** Association of statin use with severe COVID-19 outcomes.

	Statin use vs. No Statin Use Odds Ratio (95% Confidence Interval); *p*-Value		
Outcome	Adjusted for Age	Adjusted for Age, Sex, Race	Fully adjusted *
**In hospital death**	1.07 (0.49–2.32); 0.856	1.03 (0.46–2.29); 0.933	0.88 (0.37–2.08); 0.781
**ICU admission**	1.14 (0.64–2.01); 0.641	1.12 (0.62–1.99); 0.700	0.90 (0.49–1.67); 0.756
**Intubation**	0.61 (0.29–1.24); 0.175	0.57 (0.27–1.19); 0.137	0.45 (0.20–0.99); 0.048

* age, sex, race, cardiovascular disease, chronic pulmonary disease, diabetes, obesity.

**Table 3:** Association of angiotensin converting enzyme inhibitor (ACEi)/angiotensin II receptor blocker (ARB) use with severe COVID-19 outcomes *.

	ACEi/ARB use vs. No ACEi/ARB Use Odds Ratio (95% Confidence Interval); *p*-Value		
Outcome	Adjusted for Age	Adjusted for Age, Sex, Race	Fully adjusted ^†^
**In hospital death**	1.65 (0.79–3.42); 0.175	1.69 (0.79–3.57); 0.170	1.85 (0.80–4.29); 0.149
**ICU admission**	1.31 (0.77–2.23); 0.317	1.26 (0.73–2.19); 0.393	0.95 (0.52–1.75); 0.891
**Intubation**	1.40 (0.72–2.73); 0.317	1.26 (0.63-2.50); 0.502	1.00 (0.47–2.12); 0.996

* ACEi refers to angiotensin converting enzyme inhibitor. ARB refers to angiotensin II receptor blocker; ^†^ Adjusted for age, sex, race, cardiovascular disease, chronic pulmonary disease, diabetes, obesity.

## Supplementary Materials

The following are available online at https://www.mdpi.com/2076-0817/9/9/759/s1, Table S1: Statin use and in-hospital outcomes, Table S2: Angiotensin converting enzyme inhibitor (ACEi)/angiotensin II receptor blocker (ARB) use and in-hospital outcomes.

## References

[1] World Health Organization (2020). WHO Coronavirus Disease (COVID-19) Dashboard.

[2] Guan W.-J., Ni Z.-Y., Hu Y., Liang W.-H., Ou C.-Q., He J.-X., Liu L., Shan H., Lei C.-L., Hui D.S. (2020). Clinical Characteristics of Coronavirus Disease 2019 in China. N. Engl. J. Med..

[3] Huang C., Wang Y., Li X., Ren L., Zhao J., Hu Y., Zhang L., Fan G., Xu J., Gu X. (2020). Clinical features of patients infected with 2019 novel coronavirus in Wuhan, China. Lancet.

[4] Wang D., Hu B., Hu C., Zhu F., Liu X., Zhang J., Wang B., Xiang H., Cheng Z., Xiong Y. (2020). Clinical Characteristics of 138 Hospitalized Patients With 2019 Novel Coronavirus–Infected Pneumonia in Wuhan, China. JAMA.

[5] Wu Z., McGoogan J.M. (2020). Characteristics of and Important Lessons from the Coronavirus Disease 2019 (COVID-19) Outbreak in China. JAMA.

[6] Yang J., Zheng Y., Gou X., Pu K., Chen Z., Guo Q., Ji R., Wang H., Wang Y., Zhou Y.-N. (2020). Prevalence of comorbidities and its effects in patients infected with SARS-CoV-2: A systematic review and meta-analysis. Int. J. Infect. Dis..

[7] Ruan Q., Yang K., Wang W., Jiang L., Song J.-X. (2020). Clinical predictors of mortality due to COVID-19 based on an analysis of data of 150 patients from Wuhan, China. Intensive Care Med..

[8] Shi S., Qin M., Shen B., Cai Y., Liu T., Yang F., Gong W., Liu X., Liang J., Zhao Q. (2020). Association of Cardiac Injury With Mortality in Hospitalized Patients with COVID-19 in Wuhan, China. JAMA Cardiol..

[9] Wu C., Chen X., Cai Y., Xia J., Zhou X., Xu S., Huang H., Zhang L., Zhou X., Du C. (2020). Risk Factors Associated with Acute Respiratory Distress Syndrome and Death in Patients with Coronavirus Disease 2019 Pneumonia in Wuhan, China. JAMA Intern. Med..

[10] Zhou F., Yu T., Du R., Fan G., Liu Y., Liu Z., Xiang J., Wang Y., Song B., Gu X. (2020). Clinical course and risk factors for mortality of adult inpatients with COVID-19 in Wuhan, China: A retrospective cohort study. Lancet.

[11] Petrie J.R., Guzik T.J., Touyz R.M. (2017). Diabetes, Hypertension, and Cardiovascular Disease: Clinical Insights and Vascular Mechanisms. Can. J. Cardiol..

[12] Chou R., Dana T., Blazina I., Daeges M., Jeanne T.L. (2016). Statins for Prevention of Cardiovascular Disease in Adults. JAMA.

[13] Davidson M.H., Robinson J.G. (2006). Lipid-lowering effects of statins: A comparative review. Expert Opin. Pharmacother..

[14] Hong K.-S., Lee J.S. (2015). Statins in Acute Ischemic Stroke: A Systematic Review. J. Stroke.

[15] Esler M., Esler D. (2020). Can angiotensin receptor-blocking drugs perhaps be harmful in the COVID-19 pandemic?. J. Hypertens..

[16] Fang L., Karakiulakis G., Roth M. (2020). Are patients with hypertension and diabetes mellitus at increased risk for COVID-19 infection?. Lancet Respir. Med..

[17] Jarcho J.A., Ingelfinger J.R., Hamel M.B., D’Agostino R.B., Harrington D.P. (2020). Inhibitors of the Renin–Angiotensin–Aldosterone System and Covid-19. N. Engl. J. Med..

[18] Mancia G., Rea F., Ludergnani M., Apolone G., Corrao G. (2020). Renin–Angiotensin–Aldosterone System Blockers and the Risk of Covid-19. N. Engl. J. Med..

[19] Reynolds H.R., Adhikari S., Pulgarin C., Troxel A.B., Iturrate E., Johnson S.B., Hausvater A., Newman J.D., Berger J.S., Bangalore S. (2020). Renin–Angiotensin–Aldosterone System Inhibitors and Risk of Covid-19. N. Engl. J. Med..

[20] Vaduganathan M., Vardeny O., Michel T., McMurray J.J., Pfeffer M.A., Solomon S.D. (2020). Renin–Angiotensin–Aldosterone System Inhibitors in Patients with Covid-19. N. Engl. J. Med..

[21] Long B., Brady W.J., Koyfman A., Gottlieb M. (2020). Cardiovascular complications in COVID-19. Am. J. Emerg. Med..

[22] Chalmers J.D., Short P.M., Mandal P., Akram A., Hill A.T. (2010). Statins in community acquired pneumonia: Evidence from experimental and clinical studies. Respir. Med..

[23] Kwong J.C., Li P., Redelmeier N.A. (2009). Influenza Morbidity and Mortality in Elderly Patients Receiving Statins: A Cohort Study. PLoS ONE.

[24] VanderMeer M.L., Thomas A.R., Kamimoto L., Reingold A., Gershman K., Meek J., Farley M.M., Ryan P., Lynfield R., Baumbach J. (2011). Association Between Use of Statins and Mortality Among Patients Hospitalized With Laboratory-Confirmed Influenza Virus Infections: A Multistate Study. J. Infect. Dis..

[25] Vinogradova Y., Coupland C., Hippisley-Cox J. (2011). Risk of pneumonia in patients taking statins: Population-based nested case-control study. Br. J. Gen. Pract..

[26] Villarroel M.A., Blackwell D.L., Jen A. (2019). Tables of Summary Health Statistics for U.S. Adults: 2018 National Health Interview Survey.

[27] Gu Q., Paulose-Ram R., Burt V.L., Kit B.K. (2014). Prescription cholesterol-lowering medication use in adults aged 40 and over: United States, 2003–2012. NCHS Data Brief.

[28] Brett S., Myles P., Lim W.S., Enstone J.E., Bannister B., Semple M.G., Read R.C., Taylor B.L., McMenamin J., Nicholson K.G. (2011). Pre-Admission Statin Use and In-Hospital Severity of 2009 Pandemic Influenza A(H1N1) Disease. PLoS ONE.

[29] Makris D., Manoulakas E., Komnos A., Papakrivou E., Tzovaras N., Hovas A., Zintzaras E., Zakynthinos E. (2011). Effect of pravastatin on the frequency of ventilator-associated pneumonia and on intensive care unit mortality: Open-label, randomized study. Crit. Care Med..

[30] Mortensen E.M., Nakashima B., Cornell J., Copeland L.A., Pugh M.J.V., Anzueto A., Good C., Restrepo M.I., Downs J.R., Frei C.R. (2012). Population-Based Study of Statins, Angiotensin II Receptor Blockers, and Angiotensin-Converting Enzyme Inhibitors on Pneumonia-Related Outcomes. Clin. Infect. Dis..

[31] Nielsen A.G., Nielsen M.G., Riis A.H., Johnsen S.P., Sørensen H.T., Thomsen R.W. (2012). The impact of statin use on pneumonia risk and outcome: A combined population-based case-control and cohort study. Crit. Care.

[32] O’Neal H.R., Koyama T., Koehler E.A.S., Siew E., Curtis B.R., Fremont R.D., May A.K., Bernard G.R., Ware L.B., Koyama T. (2011). Prehospital statin and aspirin use and the prevalence of severe sepsis and acute lung injury/acute respiratory distress syndrome. Crit. Care Med..

[33] Papazian L., Roch A., Charles P.E., Penot-Ragon C., Perrin G., Roulier P., Goutorbe P., Lefrant J.-Y., Wiramus S., Jung B. (2013). Effect of Statin Therapy on Mortality in Patients With Ventilator-Associated Pneumonia. JAMA.

[34] Riscili B.P., Anderson T.B., Prescott H.C., Exline M.C., Sopirala M.M., Phillips G.S., Ali N. (2011). An Assessment of H1N1 Influenza-Associated Acute Respiratory Distress Syndrome Severity after Adjustment for Treatment Characteristics. PLoS ONE.

[35] Rogers A.J., Guan J., Trtchounian A., Hunninghake G.M., Kaimal R., Desai M., Kozikowski L.-A., DeSouza L., Mogan S., Liu K.D. (2019). Association of Elevated Plasma Interleukin-18 Level With Increased Mortality in a Clinical Trial of Statin Treatment for Acute Respiratory Distress Syndrome. Crit. Care Med..

[36] Yende S., Milbrandt E.B., Kellum J.A., Kong L., Delude R.L., Weissfeld L.A., Angus D.C. (2011). Understanding the potential role of statins in pneumonia and sepsis. Crit. Care Med..

[37] Castiglione V., Chiriacò M., Emdin M., Taddei S., Vergaro G. (2020). Statin therapy in COVID-19 infection. Eur. Hear. J. Cardiovasc. Pharmacother..

[38] Dashti-Khavidaki S., Khalili H. (2020). Considerations for Statin Therapy in Patients with COVID-19. Pharmacother. J. Hum. Pharmacol. Drug Ther..

[39] Fedson D.S., Opal S.M., Rordam O.M. (2020). Hiding in Plain Sight: An Approach to Treating Patients with Severe COVID-19 Infection. mBio.

[40] Goldstein M.R., Poland G.A., Graeber C.W. (2020). Are certain drugs associated with enhanced mortality in COVID-19?. QJM Intern. J. Med..

[41] Reiner Ž., Hatamipour M., Banach M., Pirro M., Al-Rasadi K., Jamialahmadi T., Radenkovic D., Montecucco F., Sahebkar A. (2020). Statins and the COVID-19 main protease: In silico evidence on direct interaction. Arch. Med. Sci..

[42] Montecucco F., Burger F., Pelli G., Poku N.K., Berlier C., Steffens S., Mach F. (2009). Statins inhibit C-reactive protein-induced chemokine secretion, ICAM-1 upregulation and chemotaxis in adherent human monocytes. Rheumatology.

[43] Jain M.K., Ridker P.M. (2005). Anti-Inflammatory Effects of Statins: Clinical Evidence and Basic Mechanisms. Nat. Rev. Drug Discov..

[44] Link A., Ayadhi T., Bohm M., Nickenig G. (2006). Rapid immunomodulation by rosuvastatin in patients with acute coronary syndrome. Eur. Heart J..

[45] Lee K., Sewa D.W., Phua G.C. (2020). Potential role of statins in COVID-19. Int. J. Infect. Dis..

[46] Teoh N., Farrell G. (2020). Statins as early therapy to mitigate COVID-19 (SARS-CoV-2)-associated ARDS and cytokine storm syndrome—Time is of the essence. J. Clin. Transl. Res..

[47] Rodrigues-Diez R., Tejera-Muñoz A., Marquez-Exposito L., Rayego-Mateos S., Sanchez L.S., Marchant V., Santamaria L.T., Ramos A.M., Ortiz A., Egido J. (2020). Statins: Could an old friend help in the fight against COVID-19?. Br. J. Pharmacol..

[48] Fedson D.S. (2013). Treating influenza with statins and other immunomodulatory agents. Antivir. Res..

[49] Fedson D.S. (2016). Treating the host response to emerging virus diseases: Lessons learned from sepsis, pneumonia, influenza and Ebola. Ann. Transl. Med..

[50] Zhang X.-J., Qin J.-J., Cheng X., Shen L., Zhao Y.-C., Yuan Y., Lei F., Chen M.-M., Yang H., Bai L. (2020). In-Hospital Use of Statins Is Associated with a Reduced Risk of Mortality among Individuals with COVID-19. Cell Metab..

[51] Rodriguez-Nava G., Trelles-Garcia D.P., Yanez-Bello M.A., Chung C.W., Trelles-Garcia V.P., Friedman H.J. (2020). Atorvastatin associated with decreased hazard for death in COVID-19 patients admitted to an ICU: A retrospective cohort study. Crit. Care.

[52] De Spiegeleer A., Bronselaer A., Teo J.T., Byttebier G., De Tré G., Belmans L., Dobson R., Wynendaele E., Van De Wiele C., Vandaele F. (2020). The effects of ARBs, ACEIs and statins on clinical outcomes of COVID-19 infection among nursing home residents. J. Am. Med Dir. Assoc..

[53] (2020). Mount Auburn Hospital. Atorvastatin as Adjunctive Therapy in COVID-19. https://clinicaltrials.gov/ct2/show/NCT4380402.

[54] London School of Hygiene and Tropical Medicine (2020). Coronavirus Response—Active Support for Hospitalised Covid-19 Patients. https://clinicaltrials.gov/ct2/show/NCT04343001.

[55] Sadeghipour P., (Rajaie Cardiovascular Medical and Research Center), Brigham and Women’s Hospital, Tehran Heart Center, Masih Daneshvari Hospital, Hazrat Rasool Hospital, Modarres Hospital, Firuzgar Hospital, Imam Khomeini Hospital (2020). Intermediate-Dose vs. Standard Prophylactic Anticoagulation and Statin vs. Placebo in ICU Patients with COVID-19. https://clinicaltrials.cov/ct2/show/NCT04486508.

[56] Kalligeros M., Shehadeh F., Mylona E.K., Benitez G., Beckwith C.G., Chan P.A., Mylonakis E. (2020). Association of Obesity with Disease Severity Among Patients with Coronavirus Disease 2019. Obesity.

[57] Sanyaolu A., Okorie C., Marinkovic A., Patidar R., Younis K., Desai P., Hosein Z., Padda I., Mangat J., Altaf M. (2020). Comorbidity and its Impact on Patients with COVID-19. SN Compr. Clin. Med..

[58] Van Walraven C., Austin P.C., Jennings A., Quan H., Forster A.J. (2009). A Modification of the Elixhauser Comorbidity Measures into a Point System for Hospital Death Using Administrative Data. Med. Care.

