# Supplementary material for: Statin Use Is Associated with Decreased Risk of Invasive Mechanical Ventilation in COVID-19 Patients: A Preliminary Study
Source: Pathogens. 2020 Sep 17;9(9):759. doi: 10.3390/pathogens9090759 (PMC7559887; doi:10.3390/pathogens9090759)
Supplement: Supplementary file 1 [file pathogens-09-00759-s001.pdf]

pathogens MDPI

Supplementary Table S1: Statin use and in-hospital outcomes.

	No Statin Use	Statin Use	p-value
**In-Hospital outcomes**			
**Need for ICU**	43 (34.1%)	50 (40.7%)	0.29
**Need for IMV**	26 (20.6%)	19 (15.4%)	0.29
**Death**	15 (11.9%)	27 (22.0%)	0.034
**ICU duration (days*)**	7.0 (3.0-14.0)	5.5 (4.0-9.0)	0.34
**IMV duration (days*)**	5.5 (2.0-13.0)	9.0 (5.0-14.0)	0.099

** Reported as median (IQR)*

Supplementary Table S2: ACEi/ARB use and in-hospital outcomes*

	No ACEi/ARB Use	ACEi/ARB Use	p-value
**In-Hospital outcomes**			
**Need for ICU**	47 (33.8%)	46 (41.8%)	0.19
**Need for IMV**	22 (15.8%)	23 (20.9%)	0.30
**Death**	16 (11.5%)	26 (23.6%)	0.011
**ICU duration (days†)**	6.00 (3.0-12.0)	6.0 (4.0-16.0)	0.67
**IMV duration (days†)**	6.00 (2.0-10.0)	7.0 (5.0-14.0)	0.17

** ACEi refers to angiotensin converting enzyme inhibitor. ARB refers to angiotensin II receptor blocker; † Reported as median (IQR)*
